# Ornamental roses for conservation of leafcutter bee pollinators

**DOI:** 10.1038/s41598-022-23041-y

**Published:** 2022-11-04

**Authors:** Palatty Allesh Sinu, Mubarak Jamal, Greeshma Shaji, M. Hariraveendra, Gopika Viswan, P. Abhiram Krishnan, Ankita Das, K. Aneha, A. R. Pooja, Spandana Salikity, V. Arathy

**Affiliations:** Central University of Kerala, Periya, Kasaragod, Kerala 671316 India

**Keywords:** Biodiversity, Conservation biology

## Abstract

Pollinator conservation is a global priority. Efforts are taken to restore pollinators by improving flower resources, a crucial driver of pollinator diversity and population growth. It helped gardening and landscaping supply chains, which introduced lists of bee-friendly plants and bee hotels, yet, desirable results seem distant. One shortcoming of the present schemes is that they lack a cohesive planning for nesting opportunities and nesting provisions for wild solitary bees, the crucial pollinators of crop and wild plants. We tested whether the world’s popular ornamental plant, rose (*Rosa chinensis* Jacq.)—a hitherto unlisted bee-friendly plant—can aid in conserving leafcutter bees, which require fresh leaves for constructing nest cells. We surveyed 2360 rose plants in 136 sites in rural and urban places and lowlands and highlands of south (8°N–12°N) and northeastern India (26°N–27°N) for the characteristic notches the bees leave on foraged leaves. We reared brood constructed with rose and non-rose leaves to examine the brood success rate. About a quarter of all the roses surveyed had the notches of leafcutter bees on the leaves. However, the proportion of cut roses varied considerably among sites. Bees used roses much higher in urban areas and lowlands than in rural areas and highlands. The selection of plants was negatively associated with pesticide application. The brood success rate was 100% for the brood that was constructed by the leaves of rose and non-rose plants. Rose flowers do not support bees, but rose leaves indeed do. We recommend rose plants in leafcutter bee conservation and restoration schemes, particularly in urban environment.

## Introduction

Pollinators are declining globally^1,2^. Many human activities have been suggested as the reasons for this precarious global phenomenon^2^; land-use change and the resulting decline of flowers and nesting opportunities have been suggested as the critical reasons^2,3^. This awareness has prompted pollinator habitat restoration efforts and pollinator conservation initiatives globally and regionally^4–7^. However, most efforts to restore pollinator habitats—agroecosystems or urban areas—have been directed towards enriching flower density and diversity^5,8^. This has opened up abooming business of bee-friendly plants and widespread use of such plants by citizen scientists and laypeople in public places and gardens^9,10^. For example, in the UK, Wyevale, a garden centre chain, fetched an annual revenue of about GBP 311 million in 2015 alone by selling bee- and pollinator-friendly plants and landscaping gardens and properties using such plants^11^. However, a study^11^ found that the lion’s share of the forb plants recommended by the garden chains is futile for attracting pollinators. This further prompted researchers to identify promising wild herbaceous plants as forb species to use in agri-environment schemes and urban landscaping^12^.

Nevertheless, restoring habitats by enhancing nesting opportunities and nesting provisions of wild pollinators received little attention^5,13,14^. It is also unclear whether any of the poor recommendations in the lists of bee-friendly plants that Garbuzov and Ratnieks^9^ and Garbuzov et al.^11^ analyzed or plants that have not even been recommended as the bee-friendly plant could be the nesting resources for wild bees.

**Figure 1:**
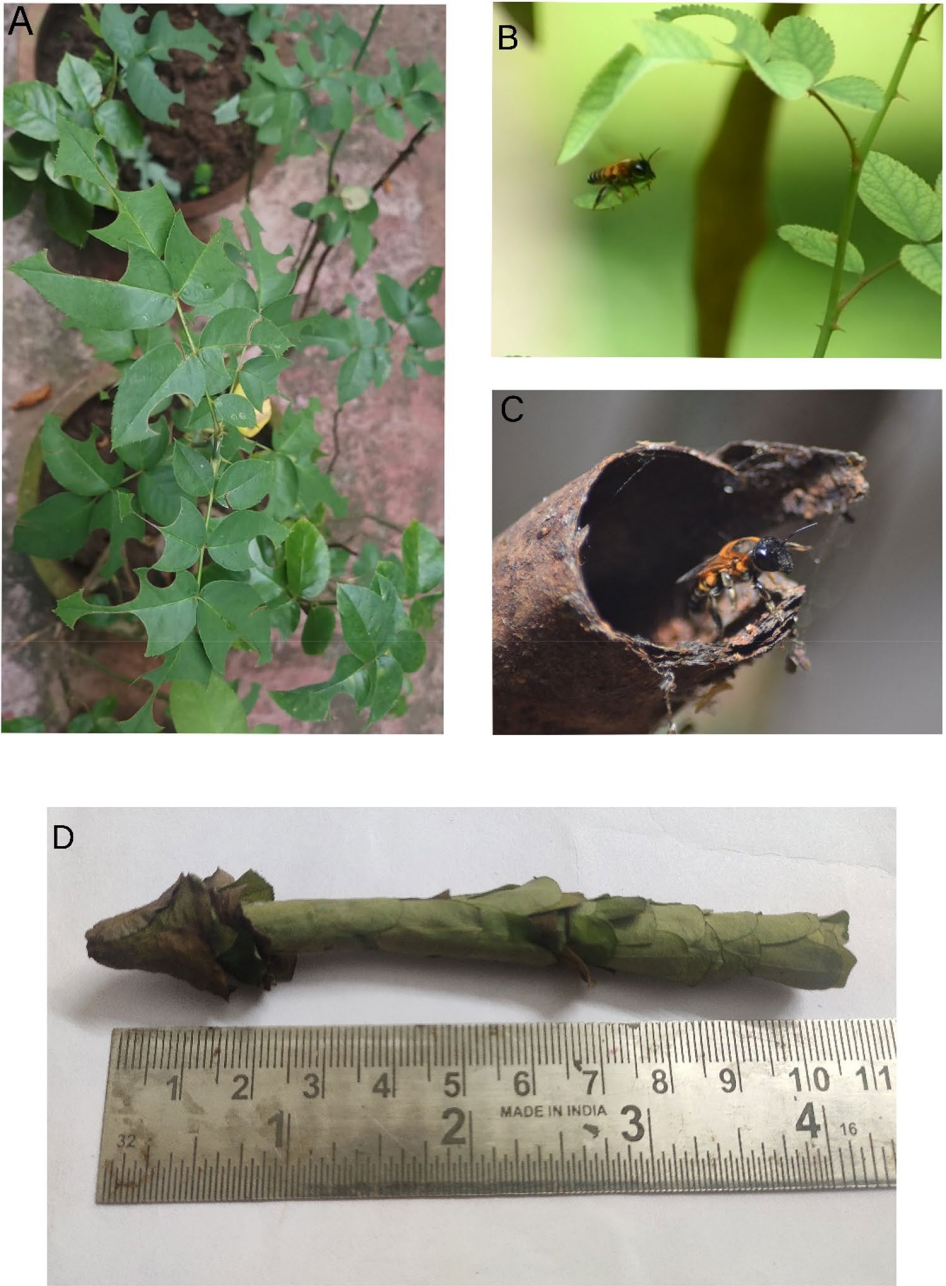
Leaf-foraging by *Megachile lanata*. (**A**) A heavily defoliated rose plant; (**B**) the bee in leaf-foraging action; (**C**) the bee at one of its natural nesting places; (**D**) two brood chambers collected from a natural nest tube. Photo credits: GV (**A**), MH (**B**) and PAK (**C**), PAS (**D**).

Several promising bee pollinators of crops require non-flower parts of plants for their biological and ecological processes^14–16^. Leafcutter bees, for instance, need young leaves for constructing brood cells^17^ (Fig. 1). Our previous studies^15–18^ suggest that the leafcutter bees prefer plants of specific lineages to forage leaf fragments. Megachilidae is a cosmopolitan solitary bee family, having over 4100 species worldwide^19^; the genus *Megachile*, which comprises leafcutters, has about half of these species and is globally distributed^19^. A recent study suggests that they can even be found at higher altitudes above 2500 m asl^20^. Many leafcutter bee species are critical pollinators of several commercial crops, such as alfalfa^21^. Some of them have been managed and introduced to countries, such as North America for managing crops, such as *Megachile rotundata*^21^.

Leafcutter bees construct nest tubes in cavities above ground^17^ (Fig. 1). This has prompted the widespread sale and use of bee hotels (= trap nests) for the management and conservation of leafcutter bees in Europe and North America, despite the absence of evidence for their effectiveness^22^. MacIvor and Packer^22^ found that the bee hotels supported fewer native leafcutter bees than they were targeted for, and a higher rate of brood parasitism. Nevertheless, bee hotels can only provide holes for constructing nest tubes. Leafcutter bees require fresh young leaves for collecting leaf discs to build brood chambers inside bee hotels or other suitable cavities. A pilot study in controlled conditions recently showed that the nesting material could drive the brood success in leafcutter bees^16^. Although we have some empirical studies for the leafcutter bees’ leaf foraging plants^15,18,23^, no recommendations have been made for the practitioners.

Recent studies^15,18^ and some studies referred therein suggest that leafcutter bees often use rose leaves to sheath the brood. The genus *Rosa* (Rosaceae: Rosid clade) has 150 species, but China rose (*Rosa chinensis* Jacq.) and its breeds, such as tea roses, have been brought worldwide as ornamental plants^24,25^. Today, over 30% of the total ornamental plant market is accounted by rose plants. They hold great symbolic and cultural value and are used for culinary and scent production purposes^26^. Leafcutter bees are often considered a major pest of rose^27–29^. They can severely defoliate plants as they return to the same leaves for more leaf cuttings (Supplementary Video S1). Often, pesticides have been recommended for managing leafcutter bees in ornamental plants^29^. Evidence suggest that leafcutter bees are more susceptible to pesticide exposure as they lack enzymes to detoxify xenobiotics^30^.

In the present research, we assessed the suitability of roses in leafcutter bee conservation initiatives. By surveying rose plants of several sites of seven latitudinal points in south India (8°N to 12°N) and northeast India (26°N to 27°N), two altitudinal ranges (at sea level to over 2000 m asl) along the Western Ghats and the central Himalayas biodiversity hotspots, and two landscape types (urban and rural), the study examined the pattern and the drivers of rose plant use by leafcutter bees. Based on the literature evidence^15,18,20^, we expected that leafcutter bees use roses in all landscapes and altitudes. By comparing the brood success of brood sheathed by the rose leaves and non-rose leaves, we tested the hypothesis that rose leaves do not hamper brood development. By surveying rose plants of different pesticide treatment (treated or untreated), we tested the hypothesis that pesticide application is negatively associated to leaf foraging.

## Materials and methods

### Study area

The study was carried out in the western coast of peninsular India along the Western Ghats Mountain chains and parts of northeast India, including the Darjeeling Himalayas. In peninsular India, the study was carried out in the state of Kerala (8°N–12°N) and Coimbatore and Ooty parts of the state of Tamil Nadu (11°N). In northeast India, the study took place in east, west, north, and south Guwahati city in Assam, parts of Siliguri, Bagdogra, Mirik, and Darjeeling hills of West Bengal (Fig. S1). In each of our major locations (latitudinal points), we surveyed the premises of several houses nested in quadrants and local sites. Together, we sampled 2360 rose plants of the same species and cultivar from 136 sites (Fig. S1). Plant use was carried out according to relevant guidelines and regulations.

### Sampling

#### Pattern and drivers of plant selection

Two thousand three hundred sixty rose plants—1954 in south India and 406 in northeastern India—were examined for the leafcutter bee-inflicted notches on the leaves (Fig. 1). The notches on the leaves imply leafcutter bee activity^15,18^. Plants with very few leaves (< 10 leaves), brought less than three months to the gardens/premises before our survey, or pruned during the study period were not considered in the present study. All the surveys were done during the peak leaf flushing and general flowering season of respective locations from November 2020 to May 2021.

The altitude, latitude, and landscape type (urban or rural) of each surveyed rose plant were recorded. Sites within the limits of municipalities and corporations (human population over one lakh) were considered urban areas; sites in villages (human population less than 10,000) were considered rural areas. Since the study was replicated at a greater spatial scale, latitude was used to represent major sites in the predictive statistical models. Sites from 8–9°N were grouped into 8°N, 9–10°N into 9°N, and so on. The height of plants, pesticide application (yes/no), leaflet length and width were collected for each sampled plant. The plants' height was used to measure the age and foliage density of plants. Leaflet length and width were considered as the measures of leaflet area. It was expected that the bees' choice of rose plants is driven by the size and density of the leaflets. Although all contributors to the present study took data on the pesticide application in their respective localities, those who did fieldwork at 8°N and 9°N took additional data on the type of pesticide used. However, this comes to about 40% of the total rose plants surveyed in South India. Those who used pesticides mentioned that they went with the recommendations of the bottles but used not more than 1% concentration. We confirmed the pesticide by verifying the bottles or the brand names of the chemicals. Accordingly, the households in our study sites used Dimethoate, Chlorpyriphos, Imidacloprid, and Botanical pesticides (brand: BioFix) to manage insect and mite pests of rose leaves and flowers. None of them were aware of leafcutter bee as a pest of rose foliage.

For the plants that have cut leaflets, fifteen first encountered leaflets were collected to measure the length and width of leaflets and cuts. Using a centimetre ruler, maximum length, maximum width, maximum cut length, and maximum cut width were measured. For plants with no cut leaflets, the middle leaflet of the first seen fifteen leaf pinnae were collected to measure the above-mentioned leaflet dimensions. The roses have an odd number of leaflets in a pinna (typically five), and the middle one is the biggest among all leaflets.

For the plants that had cut leaflets, first seen fifteen cut leaflets were collected without any pre-set conditions and counted the number of cuts on the leaflets. The number of cuts on leaflets was used to find the average number of cuts per leaflet, which was regarded as a measure of leafcutter bees’ level of reliance on the plant.

#### Brood success

We used brood that we witnessed construction at two sites (Cheruvattur (12.21°N, 75.16°E, 15 m asl) and Kanhangad (12.33°N, 75.09°E, 12 m asl)) to study brood success rate. Female *Megachile lanata* was the bee engaged in the nest construction in all those cases. We retrieved seventeen brood cells constructed using rose leaves and 31 brood cells constructed exclusively using the leaf discs of one of the following species—*Abrus* sp. (Fabaceae; N = 6), *Cassia fistula* (Fabaceae; N = 10), *Swietenia mahagoni* (Meliaceae; N = 7), and *Nephelium lappaceum* (Sapindaceae; N = 8). All the brood cells were collected from the natural nesting places of the bees—holes and cavities of laterite compound walls around gardens and house premises, metal tubes of a bicycle, metal rails of a ladder and a house balcony, crevices of doors and windows, and the PVC tubes of electric cable wires—3 days after the bees abandoned the nests, and reared at room temperature in glass beakers covered using muslin clothes. (Fig. 1). The nest sites were located at a distance of 0.8 m to 8 m from the leaf sources.

### Statistical analyses

First, we checked whether any relationship existed between the leaflet length and leaflet width of the rose leaves. That showed that they are positively related (0.54 ± 0.007, t = 69.8, P < 0.005; R^2^adjusted = 0.69; Fig. S2). Therefore, we decided to use leaflet length as a variable in the predictive statistical models.

We used Generalized Linear Model to analyze the data. We used a) cut status (binomial: cut or uncut designated by 1 and 0, respectively) and b) the average number of cuts on leaflets as the response variables in two different models to discern the drivers of plant selection and the level of dependence the bees have on roses. All the cut plants had ten or more of their leaflets with the notches inflicted by the bees. We used the One-Way ANOVA test to examine whether the leaf length differed across altitudes, latitudes, and landscape types. We used Generalized Linear Models with binomial distribution as the error type to determine whether the proportion of cut roses was influenced by altitude, latitude, landscape type, and pesticide application on the rose plants. To test whether the pesticide application drove the leaf foraging incidences, we used the data of 8°N and 9°N, where we had information on the pesticide applied to the rose plants.

In the predictive Generalized Linear Models, we used landscape type (rural vs. urban), altitude bands (< 100 m, 100–300 m, 300–600 m, 600–800 m, 800–1100 m, > 1100 m asl), sites represented by latitudes (8°N, 9°N, 10°N, 11°N, 12°N, 26°N, and 27°N), and pesticide use (used/unused) as the fixed effects, and leaflet length and plant height as covariates to understand the drivers of plant selection by the bees. In the models, binomial distribution was fitted as the error. When the number of cuts on leaves was the response variable, the subset of cut plants was used to assess the model fitness. Pesticide use and plant height were not included in the model as they were either not relevant or redundant in the model. In this Generalized Linear Model, Poisson distribution was fitted as the error. We initially included an interaction term among the predictive variables, and site was used as a random effect in the models. However, they were dropped when found their inclusion hardly improved the model's fitness.

The significance of the overall model fitness was examined using Anova available in the R-package “car”^31^. The R^2^ values of the models were calculated using the R-package “MuMIn”^32^. The fitness of the models was assessed by examining the behaviour of the residuals using the R-package DHARMa^33^. Unless mentioned otherwise, sample mean ± SD was represented as the measures of descriptive statistics, and Estimate ± SE was reported along with the model results in the entire text. All the analyses were performed in R 4.0.5^34^.

## Results

### Pattern of rose plant use by the bees

**Figure 2:**
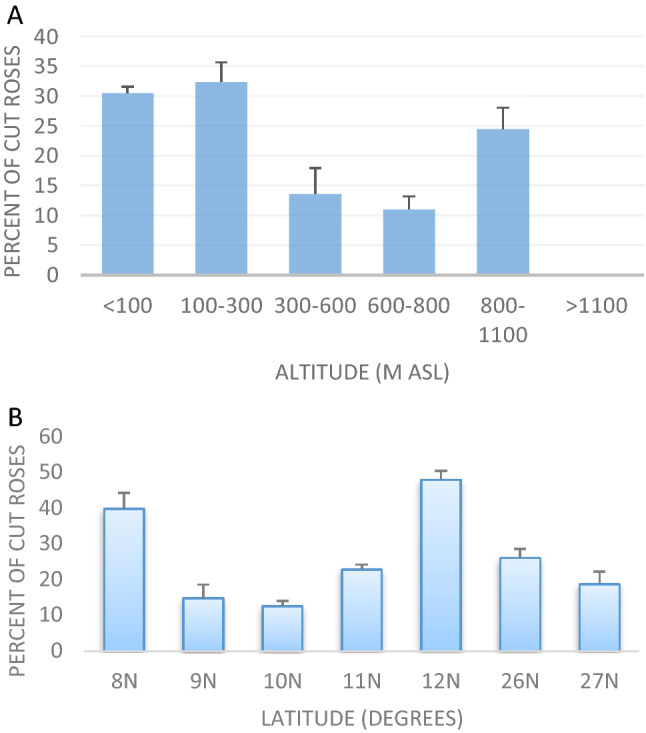
Percent of rose plants used by leafcutter bees. (**A**) The bees hardly used any rose plants in very high altitude sites; (**B**) the use of roses fluctuated across latitudinal points. mean ± SE are provided.

Out of the 2360 rose plants surveyed, 610 plants (26%) had the notches inflicted by the leafcutter bees. However, the proportion of cut plants in sites varied greatly by landscape, altitude, and latitude (Fig. 2). The proportion of cut plants in northeast India (22 ± 30%; N = 406 roses in 10 sites) and south India (29 ± 30%; N = 1954 roses in 108 sites) was not different (Estimate ± SE = 0.13 ± 0.12, Z = 0.99, P = 0.3). It suggests that the roses are perceived similarly by the bees of these two widely-separated geographical regions (mean distance between the sites of south and northeast India = 3211 km).

Seventeen percent of the total rose plants surveyed in high altitude sites (N = 362) had cuts, but in lowland sites, 31% of the surveyed rose plants (N = 1827) had cuts; roses of very high-altitude sites (> 1100 m asl) hardly had any cuts (N = 171). The difference in the proportion of cut rose plants of three altitude bands was significant (F_2,2357_ = 72.8; P < 0.005). When an average of 18% (± 30%; N = 1331) of the roses of rural sites had cut marks of the bees, an average of 38% (± 30%; N = 1029) of the roses of urban sites had the cut marks of the bees (0.93 ± 0.09, Z = 9.71, P < 0.005). Plants that had a history of pesticide treatment during the leaf flushing period were less preferred (9% ± 15%) than the plants that were never exposed to pesticides during the survey period (27% ± 26%; − 0.69 ± 0.24, Z = − 2.95, p < 0.005).

### Drivers of plant selection by the leafcutter bees

The leaflet length of the rose plants at seven latitudinal points (F5,2353 = 30.48, P < 0.005) and six altitudinal bands were different (F5,2354 = 68.94, P < 0.005). The difference was primarily due to the roses of the highest latitudinal points (27°N) and highest altitude (above 1100 m asl), where the rose leaves were large and broader. Our predictive models suggest that the probability of rose plant usage for leaf foraging was driven by pesticide application, altitude, landscape type, latitude, leaf size, and plant height (Table 1). The model with these variables in interaction terms had contributed only an additional three percent variation on the model that had the factors fitted as single effects (R^2^ = 0.89). The selection of plants was negatively affected by pesticide application on rose leaves and increasing altitude. In south India, the bee activity on rose plants was lower 800 m asl. In northeast India, the bee activity was found up to an altitude of about 1100 m asl. The bee activity was higher in the urban landscape than rural landscape. Leaf length was not a driver of the leafcutter bees' selection of rose plants. The leaf length was negatively associated with leaf-cutting by the bees (Table 1). Although pesticide application had a negative effect on leaf-cutting (F_1,795_ = 4.8, P = 0.02), the type of pesticide did not influence the bees’ decision to avoid the treated rose plants (F_3,103_ = 2.01, P = 0.11).

**Table 1:** Parameter estimates from Generalized Linear Models testing responses of rose plant usage and number of cuts on rose leaflets.

Fixed effect	Cut plants		Number of cuts/leaf	
	β [SE]	Z-value	β [SE]	Z-value
Intercept	− 0.61 [0.38]	1.6	0.12 [0.26]	0.46
Pesticide usage (used)	− 1.32 [0.29]	− 4.43**	−	−
Leaf length	− 0.20 [0.06]	− 3.18**	0.03 [0.03]	0.68
Landscape (urban)	0.93 [0.17]	5.38***	0.11 [0.08]	1.27
Plant height	1.11 [0.13]	8.51***	−	−
Altitude (100–300 m asl)	− 0.42 [0.47]	− 0.88	0.26 [0.30]	0.87
Altitude (300–600 m asl)	− 1.24 [0.41]	− 2.98**	− 0.23 [0.34]	− 0.66
Altitude (600–800 m asl)	− 1.35 [0.39]	− 3.45***	0.10 [0.19]	0.5
Altitude (800–1100 m asl)	− 5.61 [1.17]	− 4.79***	− 0.24 [0.28]	− 0.83
Altitude (> 1100 m asl)	–	–	–	–
Latitude (9°N)	− 1.34 [0.38]	− 3.51***	0.25 [0.25]	0.96
Latitude (10°N)	− 1.55 [0.27]	− 5.71***	0.39 [0.16]	2.43*
Latitude (11°N)	− 1.40 [0.25]	− 5.57***	− 0.04 [0.13]	− 0.31
Latitude (12°N)	− 0.68 [0.55]	− 1.22	0.10 [0.15]	0.61
Latitude (26°N)	0.65 [0.56]	1.15	0.10 [0.34]	0.3
Latitude (27°N)	6.42 [1.22]	5.27***	0.82 [0.35]	2.34*

The intercept is the estimate of non-pesticide plants of rural landscape, altitude below 100 m asl, and latitude 8°N. Leaf length and plant height were covariates in the model. * Indicates p < 0.05. ** Indicates p < 0.005. *** Indicates p < 0.0005.

The number of cuts per leaflet (range = 1–7) was independent of the leaflet length (0.02 ± 0.03, t = 0.43, P = 0.6). The number of cuts per leaflet was used as the proxy of the level of bees’ dependence of plants, which, however, was not driven by any of the fixed effects but latitude. Only about 6% of the total variation in the number of cuts on the leaflets was explained by the final model (Table 1).

### Brood success

**Figure 3:**
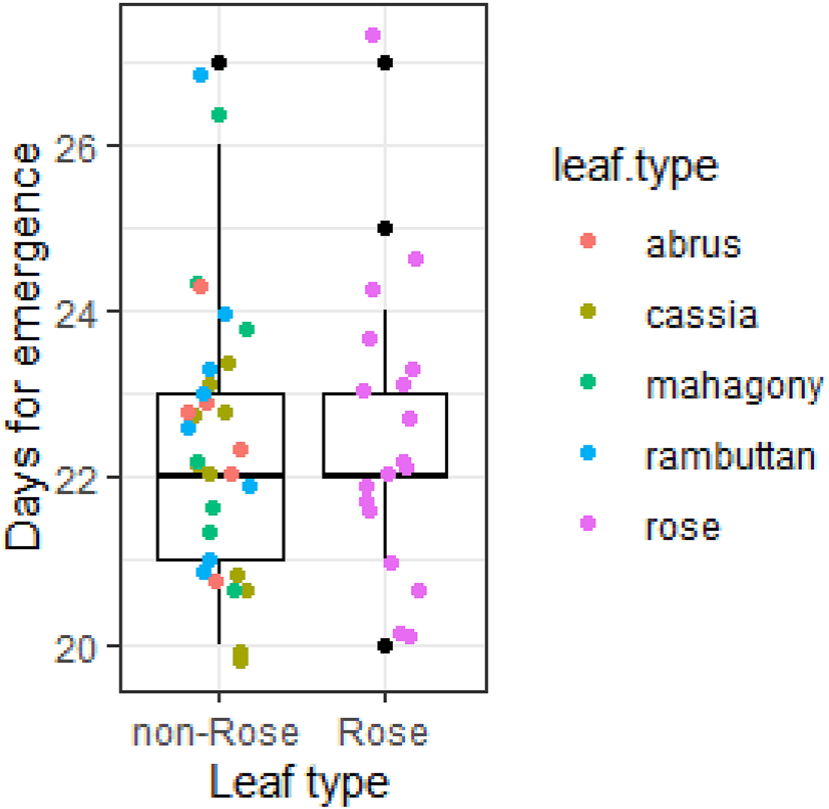
The time taken by the bees to complete development. The box plots show that the time taken by the bees of *Megachile lanata* to complete the development was not different for the brood that used rose and non-rose leaves to construct the brood cells. The non-rose leaf sources—*Abrus* sp, *Cassia fistula* (golden-shower), *Swietenia mahagoni* (mahagony), and *Nephelia lappaceum* (rambuttan)—are jittered on the box plots.

All brood cells constructed using rose leaves (N = 18) and non-rose leaves (N = 31) emerged into adult bees (F_1,47_ = 0, P = 1). Although they took 19 to 27 days to complete the development, the mean time to complete the development of brood cells constructed using the rose and non-rose leaves was not different (LM: 0.07 ± 0.48, t = 0.15, P = 0.88; Fig. 3).

## Discussion

We inquired whether one of the world’s popular ornamental plants, the rose (*Rosa chinensis* Jacq.), was also a favourite leaf foraging plant of one of the world’s most crucial solitary bee pollinator guilds, the leafcutter bees. The answer is a big yes! In our best understanding, *R. chinensis* was never listed as a bee-friendly plant as the bees hardly use their floral rewards. Our take of rose as a leaf-foraging plant brings it as a crucial bee-friendly plant. Considering the findings that the bee activity on rose leaves is higher in urban areas than rural areas, we do recommend it as an important bee plant for urban environment. However, owing to their potential as an invasive plant, we do not recommend that the ornamental roses be planted in natural areas for bee conservation. Unfortunately, rose industry perceives leafcutter bees as a pest and recommends tackling them using pesticides^27–29^.

We relied on the characteristic notches the leafcutter bees leave on leaves in our surveys^15,18^. Rose plants of all sites but above 1100 m from sea level had the leafcutter bee-foraging marks on leaflets, but the proportion of used plants varied among and between sites. The findings suggest that a range of plant-specific, landscape-specific, and plant management approaches can predict the pattern of plant use by bees. Our results indicate that (a) pesticide application can negatively influence the leaf foraging pattern of leafcutter bees in plants and (b) plants in urban places and lowlands are used better than the rural places and highlands.

There are reports that pesticides negatively affect bees^3,35^, particularly solitary bees^36,37^. However, little evidence has come for the routes bees expose to pesticides^38^. While all bees are exposed to pesticides in flowers, the leafcutter bees may be worst affected among all bees as they are likely to be exposed to pesticide-treated flowers and leaves. The households in our study sites used Dimethoate, Chlorpyriphos, Imidacloprid, and botanical pesticides (brand: BioFix) to control insect and mite pests of rose leaves and flowers. Chlorpyriphos and Dimethoate are synthetic and systemic pesticides, and Imidacloprid and BioFix have contact action. Our survey of 797 rose plants in two locations in south India discerns that the bees avoid pesticide-treated plants regardless of the pesticide type. The finding that *Megachile rotundata* has evolved no mechanism to detoxify pesticides aggravates the pesticide vulnerability of leafcutter bees^30,39^. Nevertheless, a targeted experimental study may be necessary to understand the effect of pesticide-treated leaves on the development of leafcutter bee larvae.

Bees may use roses more in urban areas if roses are predominant in the plant community in urban areas compared to other plants that the bees may prefer. Urbanization is considered a threat to biodiversity and certain biotic functions^40^. However, the response of bees to urbanization has often been illustrated positive by recent studies^41–44^, and our findings support it. Leafcutter bees are cavity nesters and use almost all unpredictable sites for constructing nest tubes^45,46^. Therefore, they are less affected by the soil, which is often polluted and compacted in urban places and agricultural landscapes^13,47^. Urban areas are likely to provide better nesting and foraging opportunities for the bees than rural places^41,48,49^. Plants belonging to Rosaceae, and *Rosa*, dominate the ornamentals in global cities^50–53^. Leafcutter bees use plants in the Rosid clade for foraging and leaf resources^15,18,23^, which are abundant in urban areas. Therefore, we expect that the urbanization may have a positive effect on leafcutter bees.

Leafcutter bees hardly used roses above 1000 m from sea level, which indicates that their distribution might be driven by the altitude, at least in India. In northern Arizona, a greater diversity of Megachilidae and *Megachile* spp. is reported 2000 m asl^20^. Southwestern USA is a heaven for the *Megachile* spp.^54^. In northeast India (Darjeeling), the senior author of the present study (PAS) surveyed roses for three years in an altitudinal band of 150 m asl to about 2000 m asl in 2018, 2019, and 2021, but found leafcutter bee damage on rose leaves for an altitude up to 1000 m asl. Our investigation of flower visitors of wild, crop, and ornamental flowering plants in the Darjeeling hills for over three years also found no bees belonging to Megachilidae above 1100 m asl. In *Amomum subulatum* (large cardamom)—a cash crop and a crucial nectar source for the bees and birds in Darjeeling and Sikkim Himalayas—*Megachile* sp was a visitor to the flowers up to an elevation of about 800 m asl^55^. Therefore, underrepresentation of leafcutter bees in high altitudes of India, rather than the rare chance that they use other leaf resources, may be a likely reason why the bees use rose plants of higher altitude sites less.

The proportion of cut rose plants varied much among sites. The present study found certain landscape and plant management factors as potential drivers for this variation. Since leafcutter bees forage young leaves for constructing brood cells, the probability of plant selection can also be driven by the availability of foraging material (pollen and nectar), the population of breeding bees, and nesting opportunities closer to leaf plants at the point of leaf flushing in leaf plants. Although we did not use them in the present investigation's statistical models, future models may find them important drivers of the pattern of leaf-cutting in plants by the bees. Natural nesting sites of leafcutter bees can be unpredictable cavities or crevices in artificial or natural materials^49,50^. Two of the authors' observations in parts of southwestern USA (PAS) and south India (PAS & AK) are that the nesting places of leafcutter bees are between 0.8 and 17 m from the leaf sources (PAS & AK, Unpublished). Therefore, the location of roses in the gardens might play a crucial role in plant use.

Leafcutter bees return to the selected plant and even the leaf for more cuttings until the leaf exhausts^15,18^ (Supplementary Video S1). This might explain why the number of cuts on leaflets was not driven by most of the variables considered in the present study. This also might explain why some rose plants in the households or sites were uncut as it is likely that the bees return to same plant for leaves.

In the present investigation, cultivars of only *R. chinensis* were considered. It may be worth mentioning that out of 114 rose plants belonging to 21 *R. chinensis* varieties and eighteen *R. banksiae* surveyed in the arboretum of the University of Arizona, 101, including 50% of *R. banksiae* had the notches of leafcutter bees (PAS & Bronstein, Unpublished). It indicates that the cultivated roses, regardless of the species and varieties, are the likely leaf foraging plants of leafcutter bees.

Leafcutter bees are cavity-nesters and critical pollinators of several cash crops, including alfalfa and pulses^21^. Installing bee hotels is a popular way of managing leafcutter bees^20^. Yet, recent meticulous field and controlled experiments have documented suboptimal propagation of the brood in bee hotels^16,22^. Studies have related this to an increasing parasitism rate in bee hotels^16,56^ and poor choice of nesting materials^16^. A recent study found 75% brood loss (N = 40 brood cells) to parasitism for *Megachile lanata* in the bee hotels in Bangalore^56^. We aimed to understand whether the leaf material affects brood success and not to understand the effect of leaf material on brood loss due to parasitism rate, which can be an exciting future study. Therefore, we collected the nest tubes immediately after the nesting activity of the bees ceased. Our findings suggest rose leaves do not hamper brood development due to possible leaf chemicals.

Leaves and petals of a range of plant species, including rose, have been unearthed in the nest tubes of leafcutter bees^15,16,18,23,57^. Plant surveys have reported an even greater number of leaf-foraging plant species in parts of southwest USA^15^ and Asia^18,58^. However, no leaf plant recommendations have been made for managing leafcutter bees in habitats. The recommended plants must meet at least two basic requirements. First, the plant should be easily manageable in habitats. Second, the nesting materials should allow brood development. As per our observations, rose meets both the criteria.

We found 100% success for brood cells sheathed by the leaves of roses, *Cassia fistula* (golden-shower), *Nephelium lappaceae* (rambuttan), *Abrus* sp., and *Swietenia mahagony* (mahagony). However, unlike other nesting resources, rose plants are easily manageable in any situation, including small households and towering apartment complexes, and are already propagated to almost all parts of the world^50–53^. Cultivated rose flowers attract hardly any bees to the flowers, so they found no place in any lists of bee-friendly plants (reference^11^ and references therein). But we recommend roses to the list of leafcutter bee-friendly plants for the first time, although it is a fact that they receive pesticides and fungicides when cultivated in an industrial scale. Although the leafcutter bees can defoliate plants considerably, no studies hitherto brought evidence to support that defoliation by leafcutter bees hampers flower output or growth in plants. It is an exciting topic for future research and may be required to alleviate the concern of ornamental plant industry such as rose industry. Although leaf is the primary resource used by the bees to construct nest tubes, some studies retrieved polyurethane materials in the leafcutter bee nests^59,60^, which the bees might have foraged under the stress of limited leaf resources. However, those studies^59,60^ did not show that they did not negatively impact the brood success^59,60^.

## Conclusion

Conservation of pollinators has been a global agenda since the IPBES report^1^ flagged the threats the pollinators and pollination services experience in the crop systems. Several studies are concerned about habitat restoration of pollinators and made recommendations for floral forb species as reports suggest that loss of foraging resources is a crucial driver of pollinator decline in the world^2,3,36^. Augmenting flower resources has become a practice in agri-environment schemes and urban landscapes of developed countries, particularly North America, Europe, and Great Britain. Though late, studies have recently been concerned about the poor quality of nesting grounds of wild pollinators due to tillage and use of agrochemicals in soils^5,13^. The present study recommends rose plants for the conservation of leafcutter bees, particularly in urban environment, and underscores that incorporating appropriate nesting resources must be an essential strategy for leafcutter bee conservation and management.

## Supplementary Information

Supplementary Information 1.

Supplementary Video S1.

## Data Availability

All data needed to evaluate the conclusions in the paper are present in the paper and the supplementary materials. The raw data can be provided by PAS pending scientific review and a completed material transfer agreement. Requests for the data should be submitted to: sinu@cukerala.ac.in.
