# Supplementary material for: Ornamental roses for conservation of leafcutter bee pollinators
Source: Sci Rep. 2022 Nov 4;12:18700. doi: 10.1038/s41598-022-23041-y (PMC9636231; doi:10.1038/s41598-022-23041-y)
Supplement: Supplementary file 1 — Supplementary Information 1. [file 41598_2022_23041_MOESM1_ESM.pdf]

Supplementary Materials for

Ornamental roses for conservation of leafcutter bee pollinators

Palatty Allesh Sinu*, Mubarak Jamal, Greeshma Shaji, M. Hariraveendra, Gopika Viswan, Abhiram Krishnan, Ankita Das, K. Aneha, A.R. Pooja, Spandana Salikity, V. Arathy

*Corresponding author. Email: sinu@cukerala.ac.in

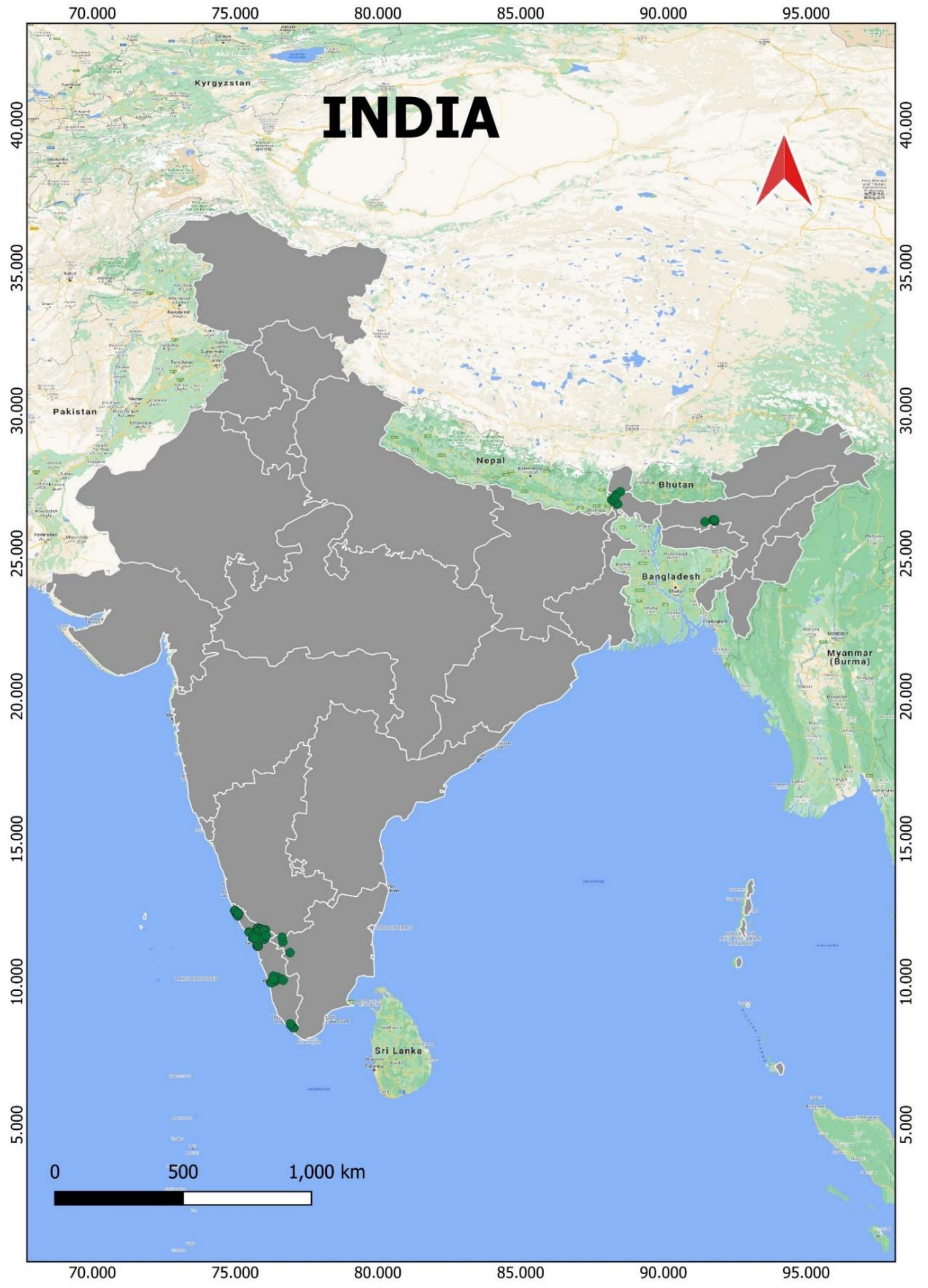

Fig. S1. Map showing study locations in south and northeast India. The map is created in Q GIS V. 3.22.1 by T.P. Rajesh

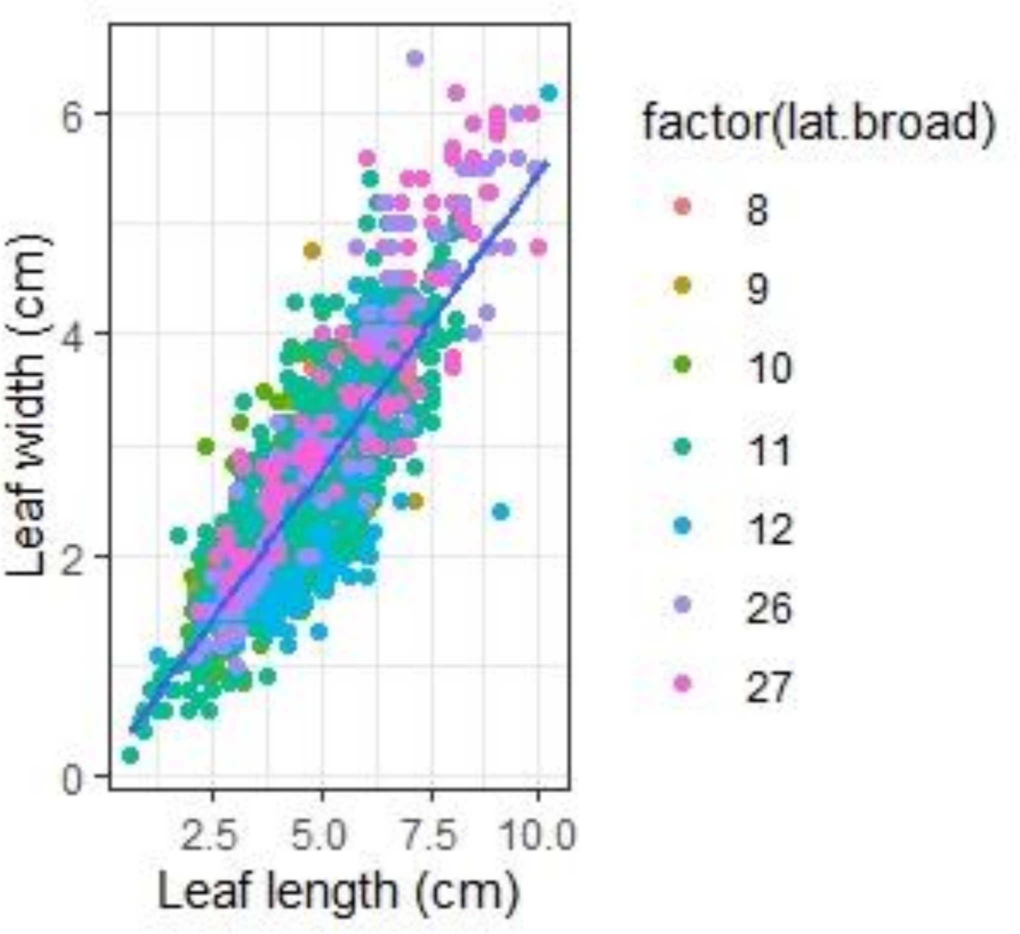

Fig. S2. Correlation between lengths and widths of the rose plants. Leaf width is positively correlated to leaf length in rose plants. Different colours represent different latitudinal points

Movie S1.
A female *Megachile lanata* is foraging leaves of rose plants
